# Supplementary material for: Chaotrope-Based Approach for Rapid In Vitro Assembly and Loading of Bacterial Microcompartment Shells
Source: ACS Nano. 2025 Mar 20;19(12):11913–23. doi: 10.1021/acsnano.4c15538 (PMC11966763; doi:10.1021/acsnano.4c15538)
Supplement: Supplementary file 1 — nn4c15538_si_001.pdf [file nn4c15538_si_001.pdf]

Supporting Information for

A chaotrope-based approach for rapid *in vitro* assembly and loading of bacterial microcompartment shells

Kyleigh L. Range[1,2†], Timothy K. Chiang[2†], Arinita Pramanik[2], Joel F. Landa[3,4], Samuel N. Snyder[5], Xiaobing Zuo[6], David M. Tiede[5], Lisa M. Utschig[5], Eric L. Hegg[3,4,9], Markus Sutter[1,7,8*], Cheryl A. Kerfeld[1,7,8,9*], and Corie Y. Ralston[2,8]

[1]MSU-DOE Plant Research Laboratory, Michigan State University, East Lansing, MI, USA, 48824, [2]Molecular Foundry Division, Lawrence Berkeley National Laboratory, Berkeley, CA, USA, 94720, [3]Cell and Molecular Biology Department, Michigan State University, East Lansing, MI, USA, 48824, [4]Molecular Plant Sciences Program, Michigan State University, East Lansing, MI, USA, 48824, [5]Chemical Sciences and Engineering Division, Argonne National Laboratory, Lemont, IL, USA, 60439, [6]X-ray Science Division, Argonne National Laboratory, Lemont, IL, USA, 60439, [7]Environmental Genomics and Systems Biology Division, Lawrence Berkeley National Laboratory, Berkeley, CA, USA, 94720, [8]Molecular Biophysics and Integrated Bioimaging Division, Lawrence Berkeley National Laboratory, Berkeley, CA, USA, 94720, [9]Department of Biochemistry and Molecular Biology, Michigan State University, East Lansing, MI, USA, 48824.

[†]These authors contributed equally to this work.

*Corresponding authors: msutter@lbl.gov and ckerfeld@lbl.gov

Keywords: Bacterial microcompartments, *in vitro*, self-assembly, urea, biotic and abiotic cargo encapsulation, catalysis, confinement

Protein	Comments	Amino acid sequence
BMC-H	Wild-type sequence	MADALGMIEVRGFVGMVEAADAMVKAAKVELIGYEK<br>TGGGYVTAVVRGDVAAVKAATEAGQRAAERVGEVV<br>AVHVIPRPHVNVDAALPLGRTPGMDKSA*
BMC-T	**6x-Histidine tag** tag on N terminus	M**HHHHHH**MDHAPERFDATPPAGEPDRPALGVLELT<br>SIARGITVADAALKRAPSLLLMSRPVSSGKHLLMMR<br>GQVAEVEESMIAAREIAGAGSGALLDELELPYAHEQ<br>LWRFLDAPVVADAWEEDTESVIIVETATVCAAIDSAD<br>AALKTAPVVLRDMRLAIGIAGKAFFTLTGELADVEAA<br>AEVVRERCGARLLELACIARPVDELRGRLFF*
$_{SpyT}$BMC-T	**6x-Histidine tag** tag on N terminus<br><br>SpyTag-001 with flanking Gly-Ser linkers	M**HHHHHH**MDHAPERFDATPPAGEPDRPALGVLELT<br>SIARGITVADAALKRAPSLLLMSRPVSSGKHLLMMR<br>GQVAEVEESMIAAREIAGAGGGSGGSAHIVMVDAYK<br>PTKGGSGGSGALLDELELPYAHEQLWRFLDAPVVA<br>DAWEEDTESVIIVETATVCAAIDSADAALKTAPVVLR<br>DMRLAIGIAGKAFFTLTGELADVEAAAEVVRERCGA<br>RLLELACIARPVDELRGRLFF*
BMC-P	**6x-Histidine tag** on C terminus	MVLGKVVGTVVASRKEPRIEGLSLLLVRACDPDGTP<br>TGGAVVCADAVGAGVGEVVLYASGSSARQTEVTNN<br>RPVDATIMAIVDLVEMGGDVRFRKDGSS**HHHHHH***
NrfA$_{SpyC}$	**StrepII tag** on C terminus<br><br>SpyCatcher-001 on N terminus	DSATHIKFSKRDEDGKELAGATMELRDSSGKTISTWI<br>SDGQVKDFYLYPGKYTFVETAAPDGYEVATAITFTV<br>NEQGQVTVNGGSGGSAPPKAEQAKIAEIPDGTIDPA<br>VWGKNYPEEYQTWKDTALPTPEGKSKYKKGNDGG<br>KVYDKLSEYPFIALLFNGWGFGIEYNEPRGHVYMMK<br>DQKEIDPSRLKGGGACLTCKTPYAPQLAQKQGVTYF<br>SQSYADAVNQIPKEHQEMGVACIDCHNNKDMGLKIS<br>RGFTLVKALDKMGVDQTKLTNQDKRSLVCAQCHVT<br>YTIPKDANMKSQDVFFPWDESKWGKISIENIIKKMRS<br>DKSYGEWTQAVTGFKMAYIRHPEFEMYSNQSVHW<br>MAGVSCADCHMPYTKVGSKKISDHRIMSPLKNDFK<br>GCKQCHSESSEWLKNQVITIQDRAASQYIRSGYALA<br>TVAKLFEMTHKQQAAGKQIDQKMYDQAKFYYEEGF<br>YRNLFFGAENSIGFHNPTEAMRILGDATMYAGKADG<br>LLRQALTKAGVDVPVKIDLELSKYTNNRGAKKLMFKP<br>EQELKDPYGPQK**WSHPQFEK***

Table S1. Amino acid sequences of the proteins used in this study.

Protein	Molecular Weight (kDa)	Extinction Coefficient (M^{-1} cm^{-1})	
		280	410
BMC-H	10.114 (monomer)<br>60.684 (tile = 6x monomers)	2980 (monomer)<br>17880 (tile)	
BMC-T	23.085 (monomer)<br>69.255 (tile = 3x monomers)	12490 (monomer)<br>37470 (tile)	
BMC-T-spyTag	25.026 (monomer)<br>75.078 (tile = 3x monomers)	13980 (monomer)<br>41940 (tile)	
BMC-P	10.910 (monomer)<br>54.55 (tile = 5x monomers)	1490 (monomer)<br>7450 (tile)	
NrfA-spyCatcher	62.499	96720	547000

Table S2. Molecular weights and theoretical extinction coefficients of the proteins used in this study.

Assembly Method – Prep #	Molar equivalents of pentamer during assembly/capping	Average # $Ru(bpy)_3$ molecules per shell	$[Ru(bpy)_3]$ in shell lumen (mM)
Combined *in vivo* assembly + *in vitro* capping – Prep 1	5x excess	49	5.8
Combined *in vivo* assembly + *in vitro* capping – Prep 2	5x excess	38	4.5
One-step *in vitro* assembly – Prep 1	1x stoichiometric amount	20	2.3
One-step *in vitro* assembly – Prep 2	1x stoichiometric amount	22	2.6

Table S3. Comparison of $Ru(bpy)_3$ cargo loading experiments by the one-step *in vitro* HTP shell assembly method and the former method combining *in vivo* assembly + *in vitro* capping, Protein concentrations were quantified by Bradford assay and ruthenium concentrations were quantified using inductively coupled plasma atomic emission spectroscopy (ICP-AES). $[Ru(bpy)_3^{2+}]$ in shell lumen value assumes that all protein mass of purified samples represents completely assembled shells with an inner diameter of 30 nm and that the encapsulated $Ru(bpy)_3^{2+}$ molecules are evenly distributed throughout all shells in the sample.

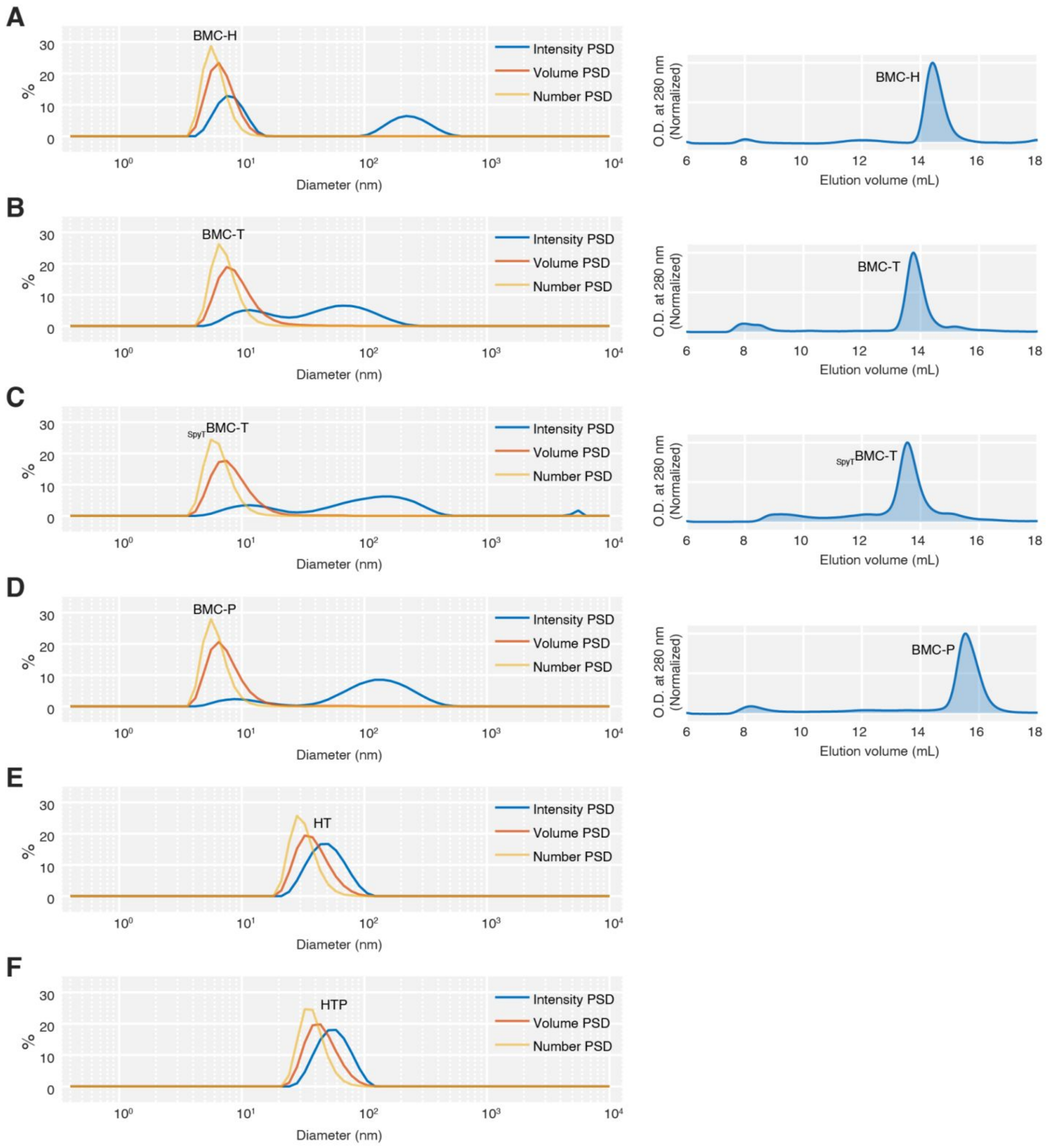

Figure S1. Purified BMC shell proteins and their assemblies are monodisperse. A–D. Typical particle size distributions (PSDs) and SEC chromatograms of purified BMC shell proteins. **E–F.** Particle size distributions of **(E)** HT minimal wiffle shells and **(F)** HTP minimal shells.

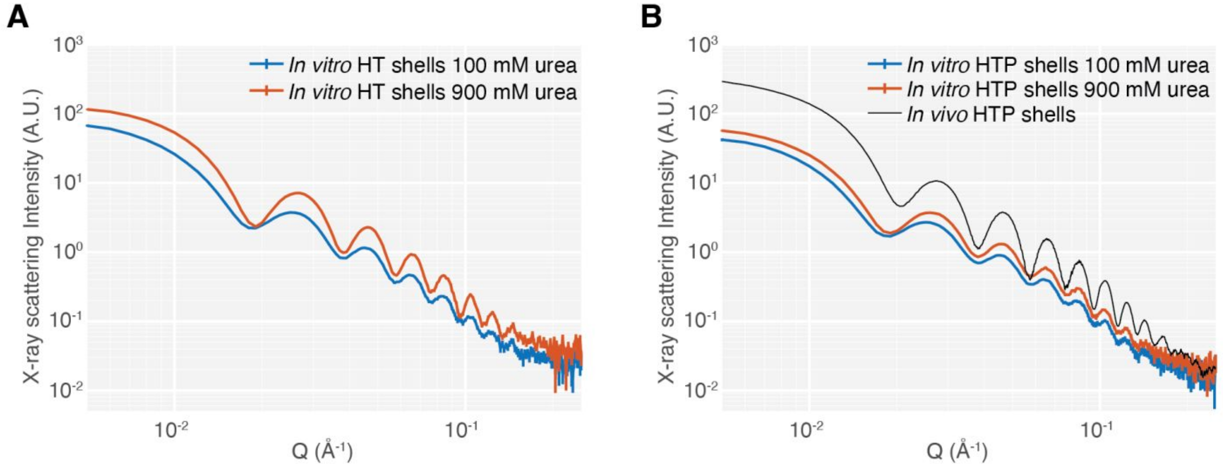

Figure S2. Experimental small-angle X-ray scattering profiles for HT and HTP shells. A. HT shells were generated *in vitro* at 100 mM and 900 mM urea. **B.** HTP shells were generated *in vitro* via a one-step assembly at 100 mM and 900 mM urea. For comparison, also shown are *in vivo* HTP shells that were heterologously expressed in *E. coli*. The profiles were offset vertically for clarity.

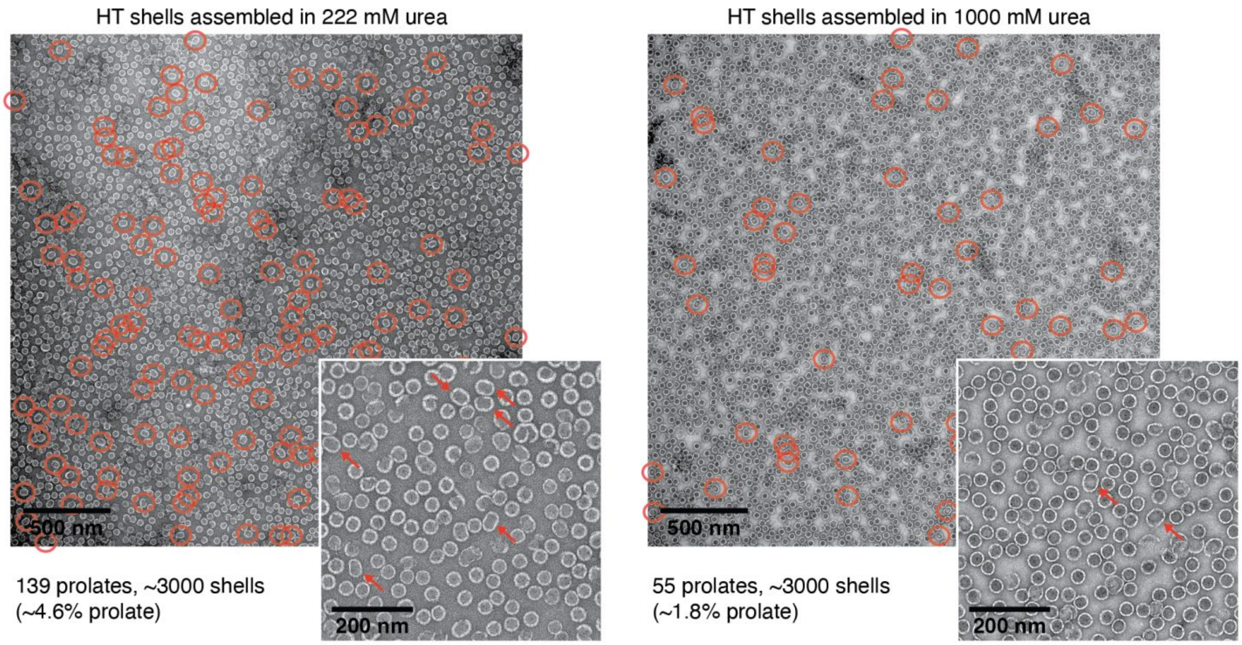

Figure S3. SEC purified HT shells assembled at low (222 mM) and high (1000 mM) urea concentrations show different numbers of prolate shells.

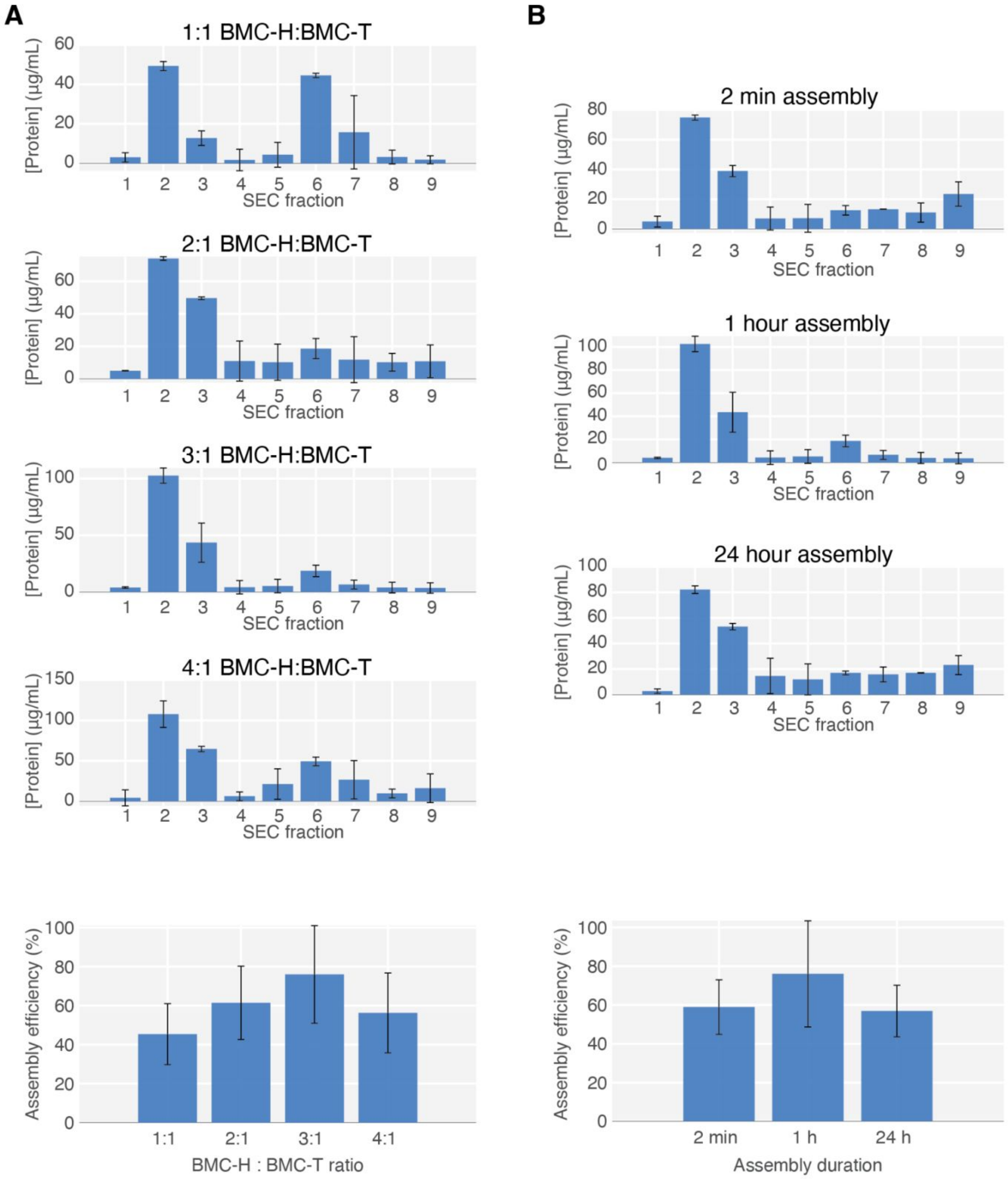

Figure S4. BCA assay quantification of protein concentrations in SEC elution fractions, and assembly efficiency calculated from BCA-measured protein concentrations. A. Protein concentrations in SEC elution fractions and assembly efficiency for assemblies in which the ratio of BMC-H:BMC-T was varied. **B.** Protein concentrations in SEC elution fractions and assembly efficiency for assemblies in which the assembly duration was varied.

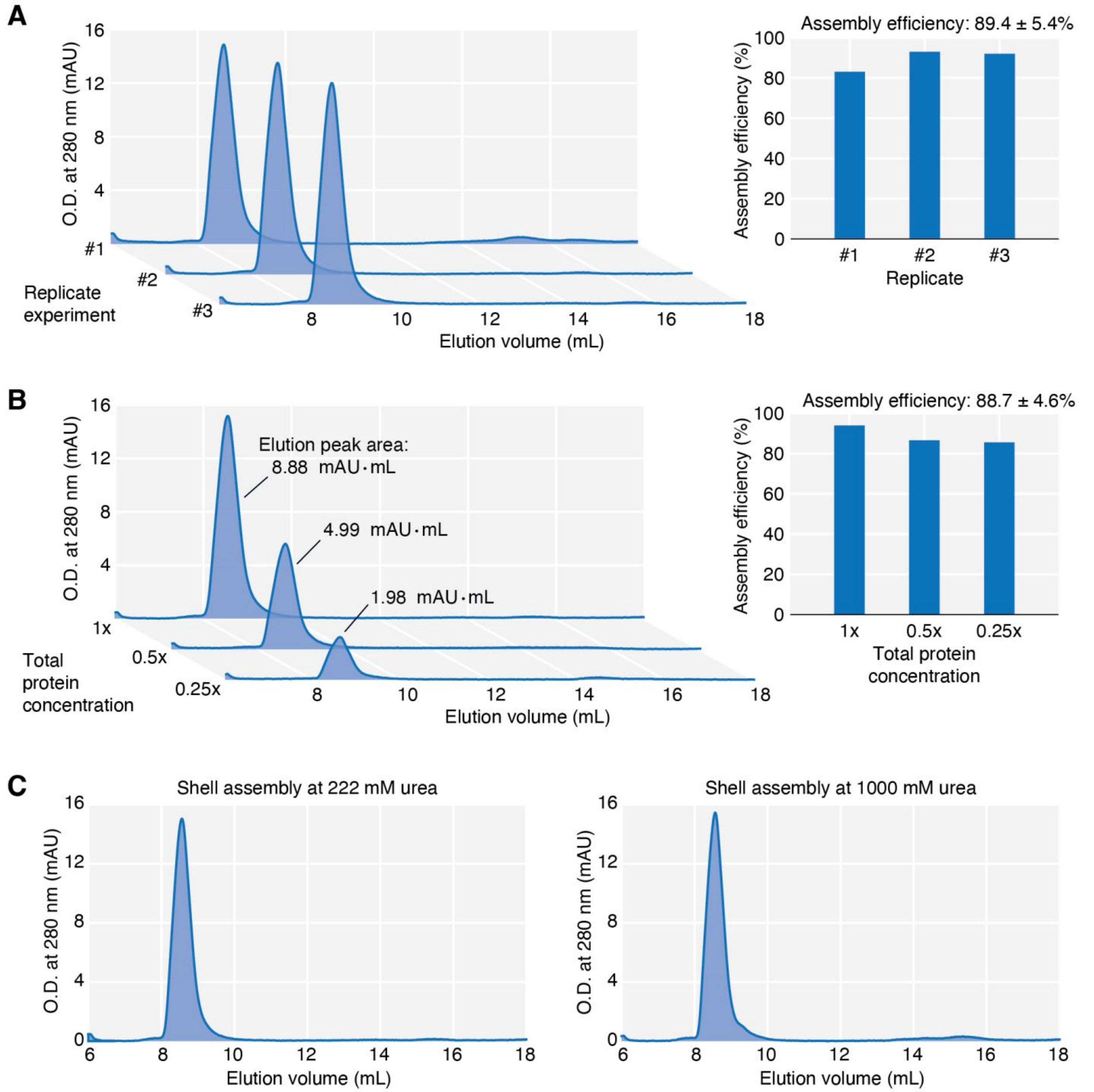

Figure S5. SEC chromatograms show that shell assembly is robust and efficient across many reaction conditions. A. Replicate reactions show reproducibly high shell assembly efficiency. These assemblies were performed as described in the main text (1 mg/mL BMC-H, 0.33 mg/mL BMC-T), with an assembly duration of 30 minutes. The replicate experiments show exceptional reproducibility, with assembly efficiencies ranging between 83–93%. **B.** Shell assembly reactions were performed at 1x total protein concentration (1 mg/mL BMC-H, 0.33 mg/mL BMC-T), at 0.5x (0.5 mg/mL BMC-H, 0.165 mg/mL BMC-T), and at 0.25x (0.25 mg/mL BMC-H, 0.0825 mg/mL BMC-T). The yield of shells (elution peak area) scaled directly with the total protein, with similar assembly efficiencies. **C.** Shell assembly in low urea (222 mM, left) and high urea (1000 mM, right).

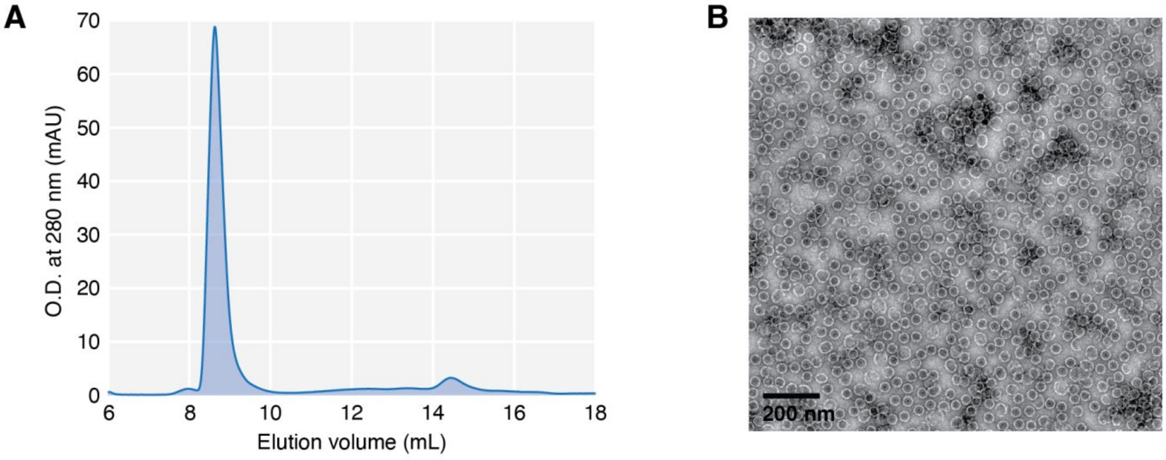

Figure S6. HT shells assembled with $_{SpyT}$BMC-T. A. SEC chromatogram of a 24-hour HT shell assembly containing 1 mg/mL BMC-H and 0.33 mg/mL $_{SpyT}$BMC-T. **B.** TEM micrograph of HT_{SpyT} shells.

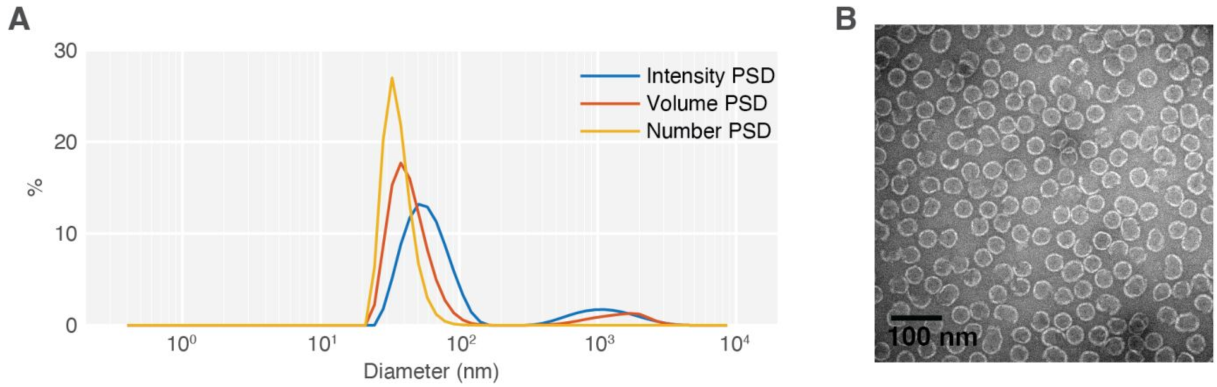

Figure S7. NrfA-loaded HT shells. A. DLS particle size distributions of SEC purified $NrfA_{SpyC}$-loaded HT shells. **B.** TEM micrograph of $NrfA_{SpyC}$-loaded HT shells.

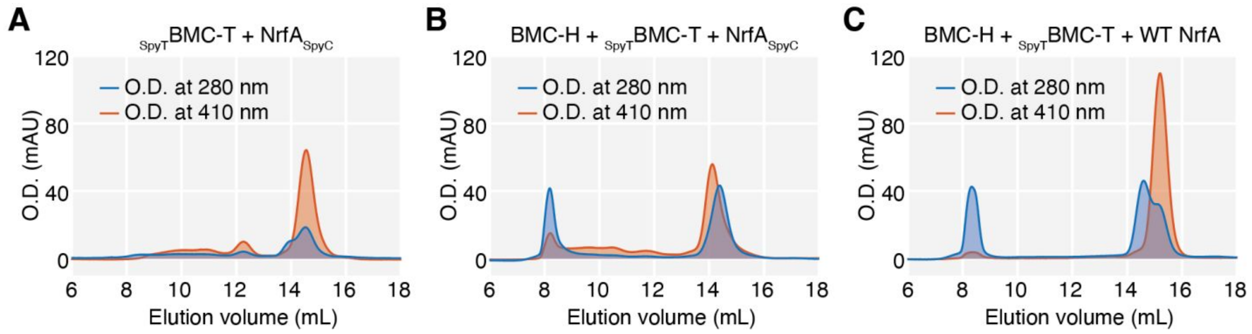

Figure S8. A. SEC chromatogram of a control reaction containing only ${}_{SpyT}$BMC-T–NrfA$_{SpyC}$ conjugation (no BMC-H added) shows no elution of protein aggregation in the void volume. **B–C.** SEC chromatograms of an HT shell assembly with ${}_{SpyT}$BMC-T and (**B**) NrfA$_{SpyC}$ and (**C**) wild-type NrfA.

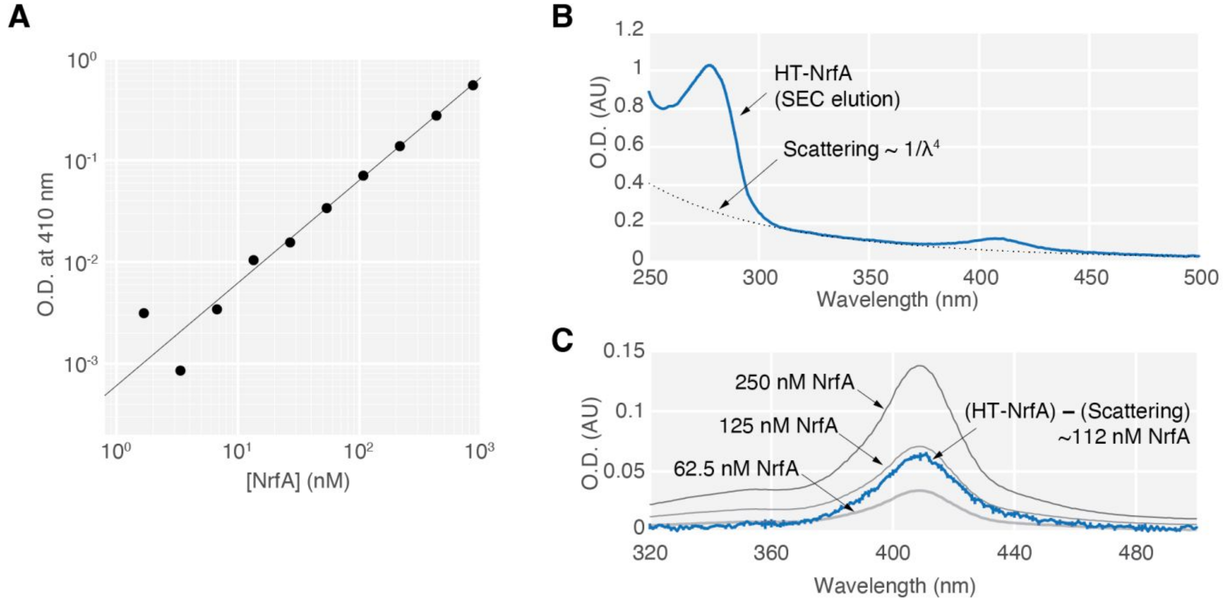

Figure S9. UV-Vis quantification of NrfA concentration in SEC-purified HT shells. A. O.D. 410 nm standard curve for serial dilutions of NrfA$_{SpyC}$ stocks with known concentrations. **B.** UV-Vis spectrum for NrfA$_{SpyC}$-loaded HT shells. The dotted line shows a model representing the contribution to the total extinction from scattering. **C.** Enzyme concentration is quantified by isolating the heme Soret absorbance peak at 410 nm. Spectra of several NrfA$_{SpyC}$ solutions with known concentrations are shown for comparison.

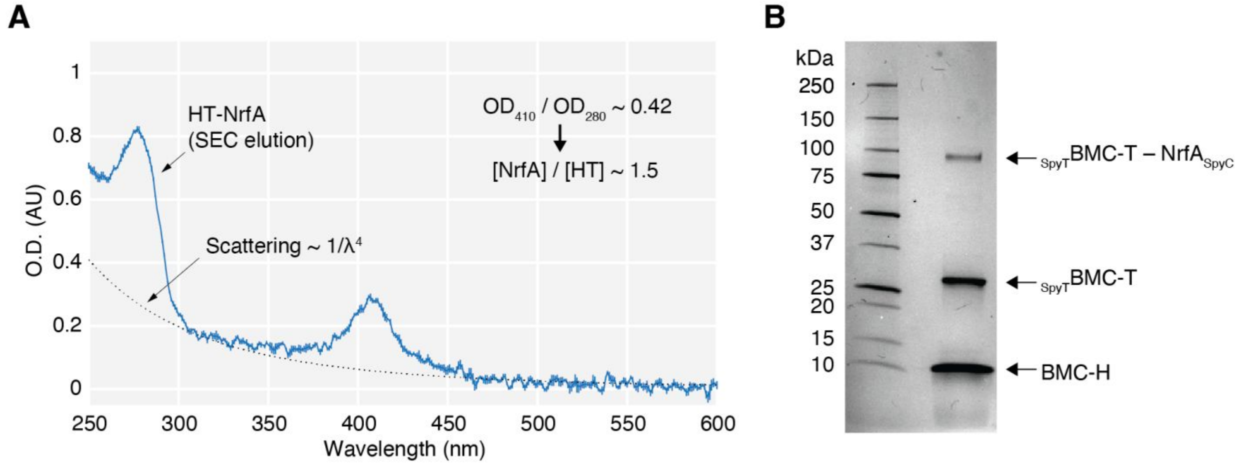

Figure S10. Changing reaction conditions results in higher shell loading efficiency. A. UV-Vis spectrum for SEC-purified $NrfA_{SpyC}$-loaded HT shells that were assembled with an excess amount of enzyme cargo. The loading efficiency is estimated to be approximately 1.5 enzymes per shell. **B.** SDS-PAGE analysis shows that enzymes loaded in the shells are conjugated to ${}_{SpyT}$BMC-T tiles.
